# Analysis of epidemiological characteristics of four natural-focal diseases in Shandong Province, China in 2009-2017: A descriptive analysis

**DOI:** 10.1371/journal.pone.0221677

**Published:** 2019-08-27

**Authors:** Rui Chen, Zengqiang Kou, Liuchen Xu, Jie Cao, Ziwei Liu, Xiaojing Wen, Zhiyu Wang, Hongling Wen

**Affiliations:** 1 Department of Microbiological Laboratory Technology, School of Public Health, Shandong University, Key laboratory for the prevention and control of infectious diseases (key laboratory of China’s “13th Five-Year”, Shandong University), Jinan, Shandong Province, China; 2 Shandong Center for Disease Control and Prevention, Shandong Provincial Key Laboratory of Infectious Disease Prevention and Control, Jinan, Shandong Province, China; Georgia Southern University Jiann-Ping Hsu College of Public Health, UNITED STATES

## Abstract

**Background:**

Natural-focal diseases are serious diseases that endanger human health. They threaten about 100 million people in Shandong Province, and cause illness in thousands of people each year. However, information on the epidemiological characteristics of natural-focal diseases in Shandong Province has been limited. The purpose of the study was to describe and analyze the epidemiological characteristics of natural-focal diseases in Shandong Province, 2009–2017.

**Methods:**

We describe the incidence and distribution of four natural-focal diseases in Shandong Province using surveillance data from 2009–2017.

**Results:**

From 2009–2017, 11123 cases of four natural-focal diseases including 257 deaths were reported in Shandong Province, China. The four natural-focal diseases were severe fever with thrombocytopenia syndrome (SFTS), human granulocytic anaplasmosis (HGA), typhus, and scrub typhus. The high-risk groups of the four diseases were farmers and the elderly. The incidence rate of scrub typhus was significantly higher in females. However, this difference was not seen in the other three diseases. The four diseases were mainly clustered in the middle-southern part of Shandong Province and the Shandong Peninsula. The annual incidence of SFTS and scrub typhus increased, typhus was relatively stable, and HGA declined. However, the range of SFTS expanded, while HGA shrunk, and typhus and scrub typhus were unchanged. The epidemic period of SFTS and HGA was from May to October, typhus was from October to November, and scrub typhus was from September to November. The fatality rates of SFTS, typhus, scrub typhus, and HGA were 9.19%, 0%, 0.01%, and 2.24%, respectively.

**Conclusions:**

Our study described and analyzed the prevalence of natural-focal diseases in Shandong Province, and confirmed that age was closely related to the SFTS fatality rate. This study may help to improve the understanding of the prevalence of natural-focal diseases in Shandong Province in recent years and to better develop accurate prevention and control strategies for natural-focal diseases.

## Introduction

Natural-focal diseases are infectious diseases that are circulated by pathogens in natural host animals, and can infect people when they enter the natural epidemic focus. Natural-focal diseases are a large group of diseases. At present, there are more than 180 kinds of natural-focal diseases in the world, including viral diseases, bacterial diseases, rickettsiosis, chlamydia, spirochetes, fungal diseases, protozoa diseases and other parasitic diseases. However, in recent years, a variety of old natural-focal diseases have revived, such as Zika virus disease, and dengue fever (DF). In addition, new pathogens of natural-focal diseases have constantly been found in China, such as severe fever with thrombocytopenia syndrome phlebovirus (SFTSV), *Candidatus Rickettsia tarasevichiae*, and *Rickettsia sibirica subspecies sibirica* BJ-90 [1–3]. In the past 55 years, 8,350,754 cases of natural-focal diseases involving 24 types of natural-focal diseases were reported in Chinese journals [4]. Natural-focal diseases constitute a serious threat to public health. Shandong Province is the second most populous province in China with a population of about 100 million, of which about 40% live on agricultural land. The climate in Shandong Province is warm with four distinct seasons and the terrain is diverse, which provides favorable conditions for the occurrence of many natural-focal diseases. Therefore, we chose Shandong Province as a research site for natural-focal diseases. In the capital Jinan, there were 1248 reported cases of 9 different natural-focal diseases in 2004–2013 [5]. However, only limited information concerning the epidemiologic characteristics of natural-focal diseases in Shandong Province is available, especially for recent years. Therefore, it is necessary to describe the epidemic characteristics of the natural-focal diseases in Shandong Province in recent years.

We selected four natural-focal diseases which had great impacts on the human health in Shandong Province. The four natural-focal diseases are HGA, typhus, SFTS, and scrub typhus, and are transmitted through insect vectors. SFTS and HGA are emerging natural-focal diseases in Shandong Province which are closely related to each other and have high fatality rates. Typhus and scrub typhus are two natural-focal diseases with high morbidity in local areas. The four natural-focal diseases were monitored intensively by the local health departments because of their great threat to local residents. The pathogens of SFTS and HGA are SFTSV and *Anaplasma phagocytophilum* (AP), respectively, and the main vectors are ticks [6]. Typhus, including epidemic typhus and endemic typhus, is caused by Typhus group Rickettsia. Typhus in Shandong Province is mainly endemic typhus [7]. Endemic typhus is caused by *Rickettsia typhi* and is mainly transmitted by *Xenopsyllae cheopis*, as rodents are their natural hosts. Scrub typhus is caused by the intracellular pathogen *Orientia tsutsugamushi* and is transmitted by chigger mites [8]. We described the magnitude and distribution of these diseases in Shandong Province based on the notifiable reporting dates, focusing on a three-dimensional distribution from 2009 to 2017 and the characteristics of SFTS fatality. SFTS was not monitored in China until 2010, and hence there was no monitoring data for SFTS in 2009. There were few reported HGA cases in Shandong Province in recent years, and HGA was not monitored in 2017.

## Methods

### Study site

**Fig 1:**
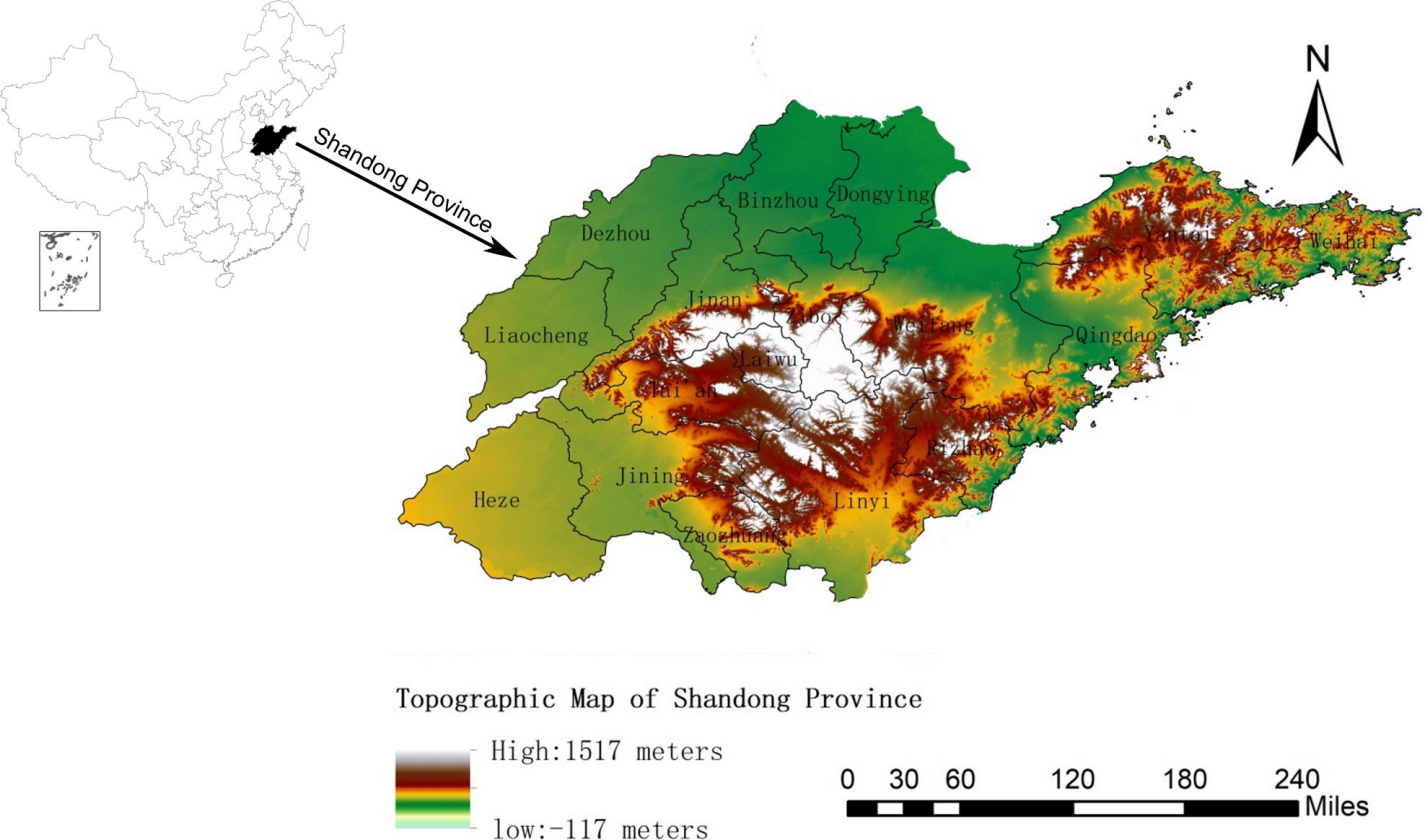
Topographic map of Shandong Province.

Shandong Province is located on the eastern coast of China (longitude 114°19’ E to 122°43’ E, latitude 34°22’ N to 38°23’ N), with an area of 157,000 km^2^, of which the vegetation coverage is about 77.54%, the planted land area is about 53.82%, the mountain area is about 14.59%, and the hilly area is about 15.39%. It is mountainous in the middle-southern part of Shandong Province and the Shandong Peninsula but mostly flat and hilly on the periphery. It is on the lower reaches of the Yellow River, and extends out in to the Pacific Ocean in the form of the Shandong Peninsula, with a coastline of 3,121 km (Fig 1). Shandong Province has a monsoon climate of medium latitudes (the average annual temperature is 13.6–14.3°C, and the average annual precipitation is 543–845 mm).

### Case definition

Since January 1, 2004, China has used the direct network reporting system of the China Infectious Disease Reporting Information System. The four natural-focal diseases cases were diagnosed according to the diagnostic criteria issued by the health department of China (S1 File).

### Data collection

Data of the four diseases were collected through passive reporting. SFTS, scrub typhus and HGA are emerging natural-focal diseases in Shandong Province. The cases of these diseases are mainly reported by local hospitals through the Shandong Disease Reporting Information System (SDRIS), and the provincial CDC is responsible for diagnosis and review. As a natural-focal disease that has existed for a long time in Shandong Province, typhus is reported directly by the township/town hospital through SDRIS, and the county-level CDC is responsible for diagnosis and review. Information of the four natural-focal diseases cases includes gender, age, occupation, residential address, date of illness onset, and outcome of the illness comes from SDRIS. The infectious disease report card was exported according to the date of onset. The population data for 2009–2017 of Shandong Province came from the Shandong Province Bureau of Statistics.

### Data analysis

The age distribution, gender distribution, occupation distribution, seasonal distribution, and regional distribution of the cases were summarized using Excel 2010, and IBM SPSS Statistics 24.0 (online) was used to perform the statistical analysis. The different rates were analyzed using the *χ*^*2*^ test. The trend test was analyzed using the Mantel-Haenszel test of trends [9, 10]. All tests were 2-tailed and statistical significance was set at *P* < 0.05. The base diagrams of Shandong Province were from the Resource and Environment Data Cloud Platform. Software ArcGis10.2 was used to plot the topographic map of Shandong Province and the geographical distribution of cases.

### Ethical approval

It was determined by the National Health and Family Planning Commission, China, that the data collection for natural-focal diseases cases was part of the continuing public health surveillance system of notifiable infectious diseases in China and was exempt from the institutional review board assessment.

## Results

### SFTS

From 2010 to 2017, a total of 2731 confirmed cases of SFTS including 251 deaths in Shandong Province were reported to SDRIS.

#### Time distribution

**Fig 2:**
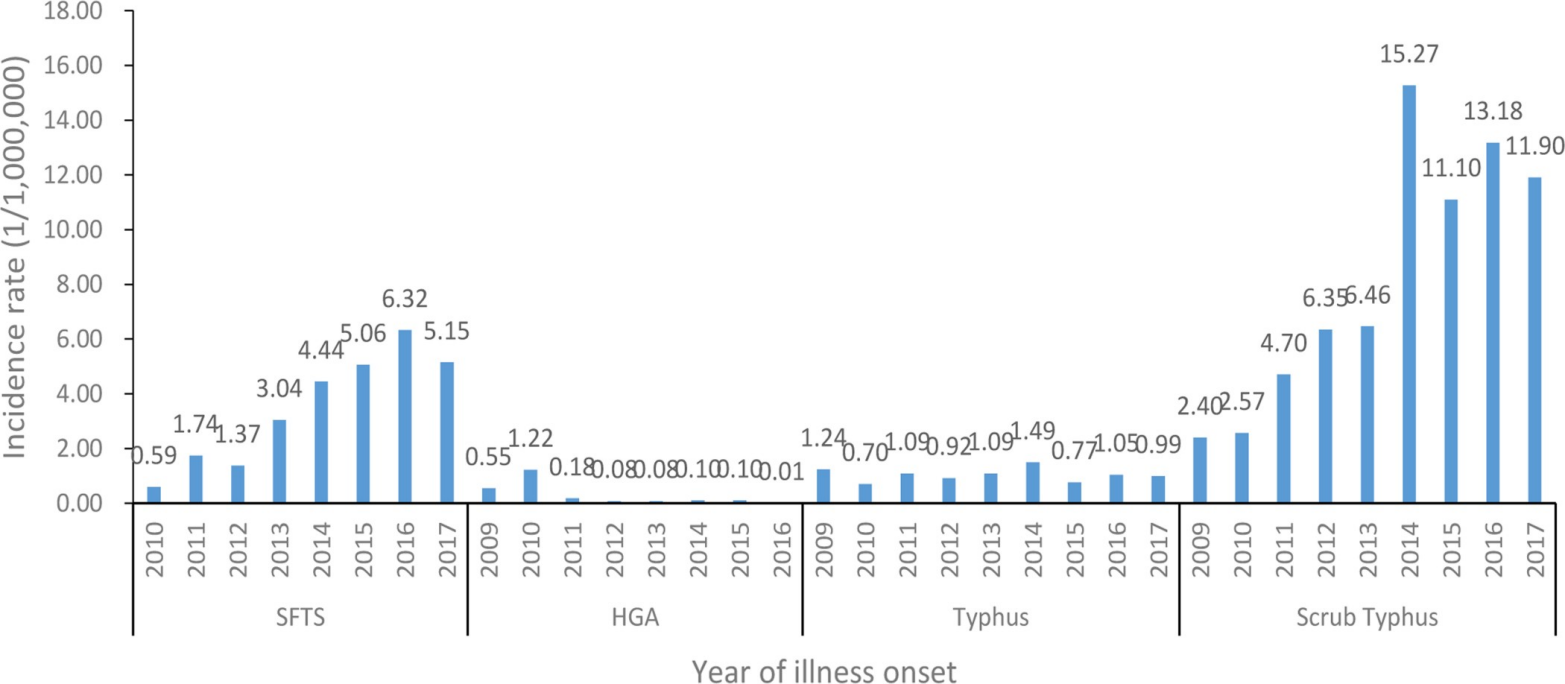
During 2009–2017, the morbidity of the four natural-focal diseases in Shandong Province.

**Fig 3:**
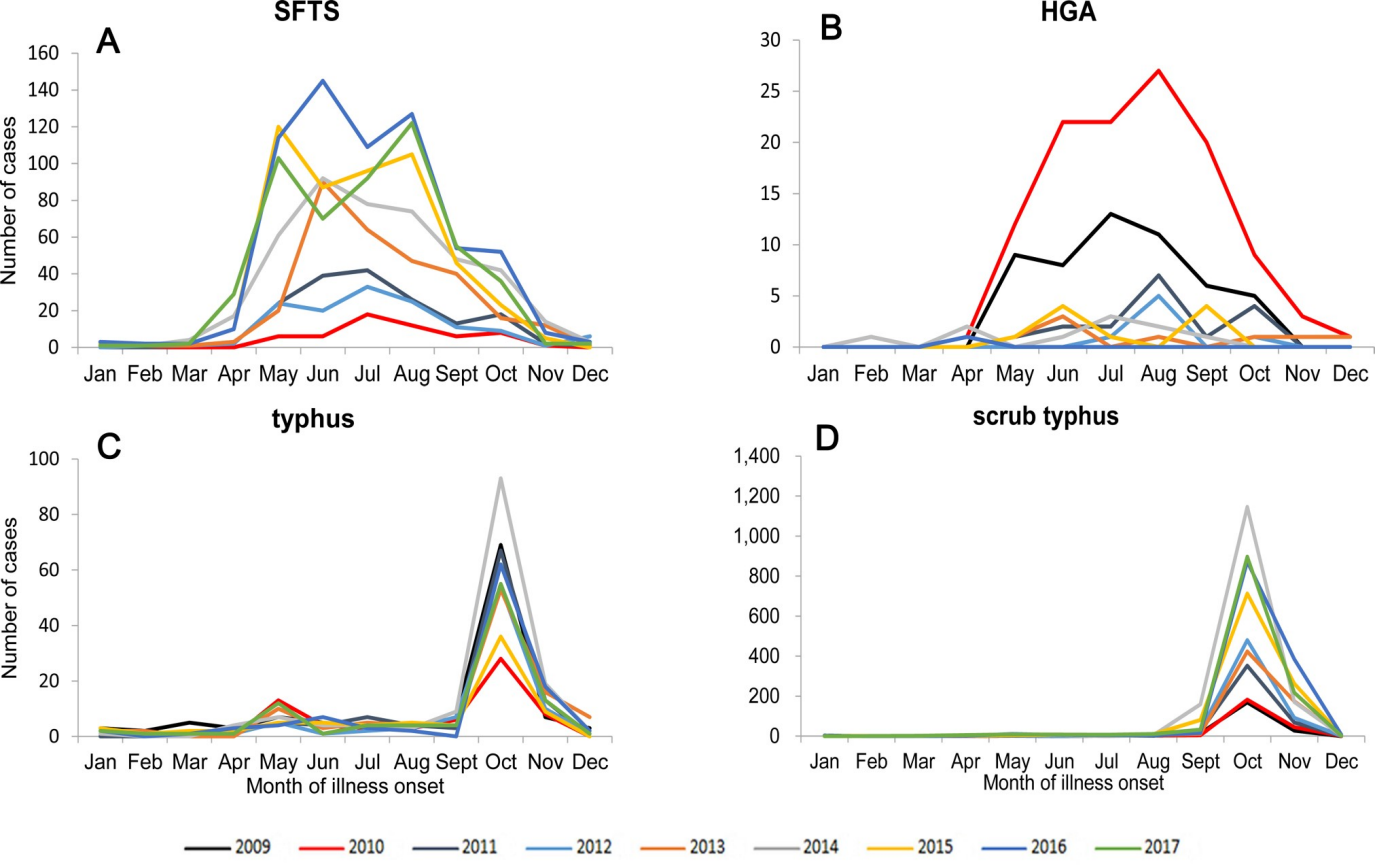
During 2009–2017, the aggregate number of cases by month in Shandong Province. (A): The aggregate number of SFTS cases by month from 2010 to 2017. (B): The aggregate number of HGA cases by month from 2009 to 2016. (C): The aggregate number of typhus cases by month from 2009 to 2017. (D): The aggregate number of scrub typhus cases by month from 2009 to 2017.

An average of 3.47 cases per one million residents were reported each year in Shandong Province during 2010–2017, with the highest recorded in 2016 (6.32 cases/1,000,000) and the lowest in 2010 (0.59 cases/1,000,000) (Fig 2). The incidence of SFTS showed obvious seasonal characteristics. During 2010–2017, 94.03% (2568/2731) of SFTS cases were reported from May to October, and the peaks of reported SFTS cases from 2010 to 2017 occurred in the summer and autumn, July, July, July, June, June, May, June, August, respectively (Fig 3A; Table 1).

**Table 1:** Demographic and epidemiological characteristics of the four natural-focal diseases in Shandong Province, 2009–2017.

	SFTS (2010–2017)		HGA (2009–2016)		Typhus (2009–2017)		Scrub typhus(2009–2017)	
	No. of cases	Proportion(%)	No. of cases	Proportion(%)	No. of cases	Proportion(%)	No. of cases	Proportion(%)
Age group (years)								
0-	3	0.11	1	0.45	33	3.63	175	2.41
10-	15	0.55	0	0.00	34	3.74	87	1.20
20-	45	1.65	3	1.35	39	4.29	191	2.63
30-	77	2.82	13	5.83	61	6.71	380	5.23
40-	297	10.88	34	15.25	153	16.83	1100	15.15
50-	710	26.00	50	22.42	230	25.30	2061	28.39
60-	859	31.45	58	26.01	214	23.54	1917	26.40
70-	561	20.54	54	24.22	114	12.54	1028	14.16
>80	164	6.01	10	4.48	31	3.41	321	4.42
Total	2731	100.00	223	100.00	909	100.00	7260	100.00
Gender								
Male	1384	50.68	114	51.12	482	53.03	3148	43.36
Female	1347	49.32	109	48.88	427	46.97	4112	56.64
Occupation								
Children	3	0.11	0	0.00	22	2.42	146	2.01
Students	14	0.51	1	0.45	40	4.40	106	1.46
Laborers	51	1.87	7	3.14	29	3.19	166	2.29
Farmers	2349	86.01	186	83.41	773	85.04	6420	88.43
Retirees	77	2.82	12	5.38	14	1.54	120	1.65
Others	237	8.68	17	7.62	31	3.41	302	4.16
Total	2731	100.00	223	100.00	909	100.00	7260	100.00
Season								
Spring	94	3.44	6	2.69	34	3.74	21	0.29
Summer	1553	56.87	106	47.53	137	15.07	110	1.52
Autumn	1015	37.17	105	47.09	592	65.13	5653	77.87
Winter	69	2.53	6	2.69	146	16.06	1476	20.33
Month								
Jan	8	0.29	0	0.00	17	1.87	9	0.12
Feb	8	0.29	2	0.90	7	0.77	3	0.04
Mar	13	0.48	0	0.00	11	1.21	6	0.08
Apr	73	2.67	4	1.79	16	1.76	12	0.17
May	472	17.28	24	10.76	68	7.48	47	0.65
Jun	549	20.10	40	17.94	34	3.74	34	0.47
Jul	532	19.48	42	18.83	35	3.85	29	0.40
Aug	538	19.70	53	23.77	31	3.41	48	0.66
Sept	273	10.00	32	14.35	44	4.84	370	5.10
Oct	204	7.47	20	8.97	517	56.88	5235	72.11
Nov	45	1.65	4	1.79	113	12.43	1442	19.86
Dec	16	0.59	2	0.90	16	1.76	25	0.34

#### Population distribution

**Fig 4:**
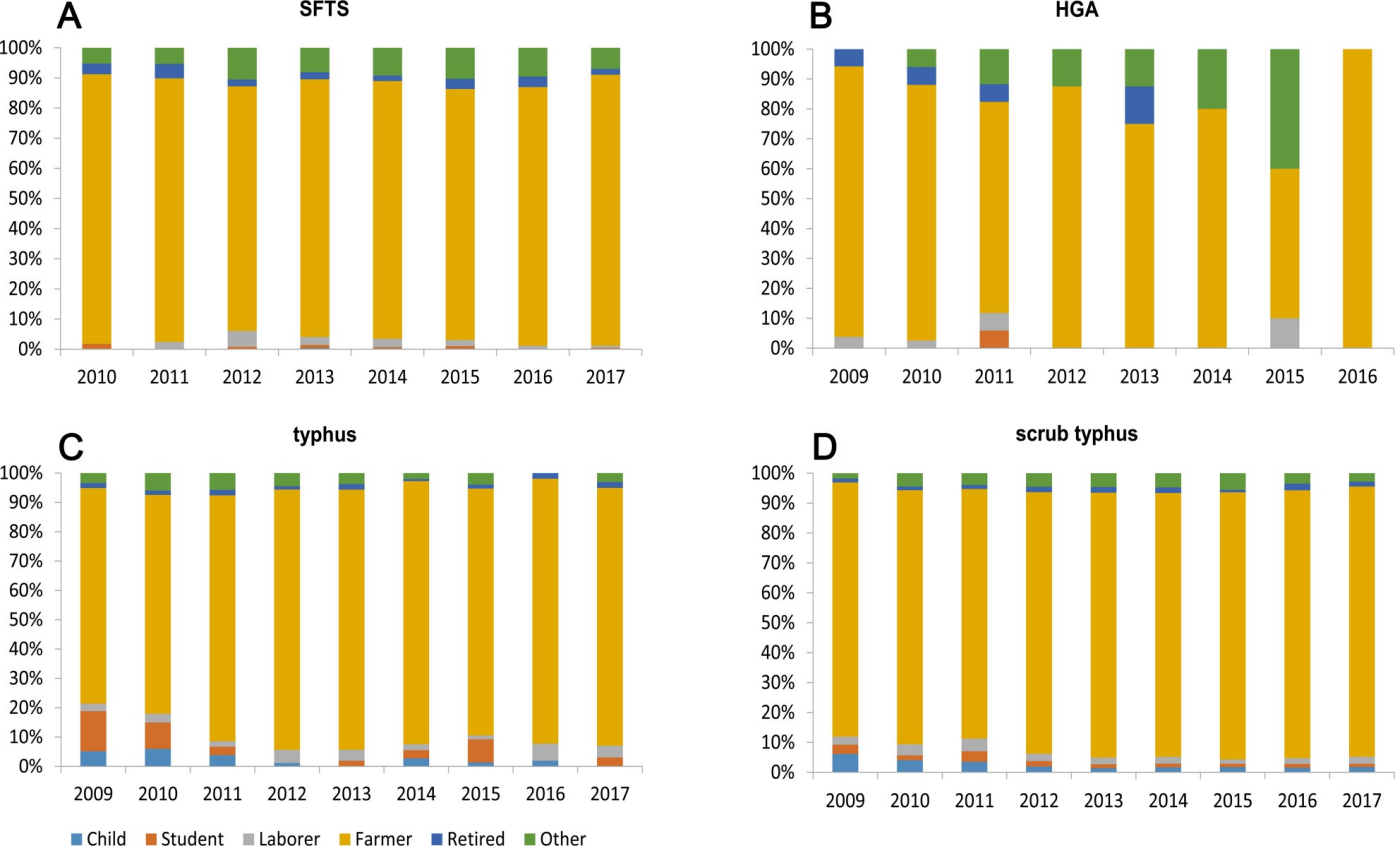
The proportion of different occupations of the four natural-focal diseases cases by year, 2009–2017. (A): Proportion of different occupations in SFTS cases by year in Shandong Province. (B): Proportion of different occupations in HGA cases by year in Shandong Province. (C): Proportion of different occupations in typhus cases by year in Shandong Province. (D): Proportion of different occupations in scrub typhus cases by year in Shandong Province.

**Fig 5:**
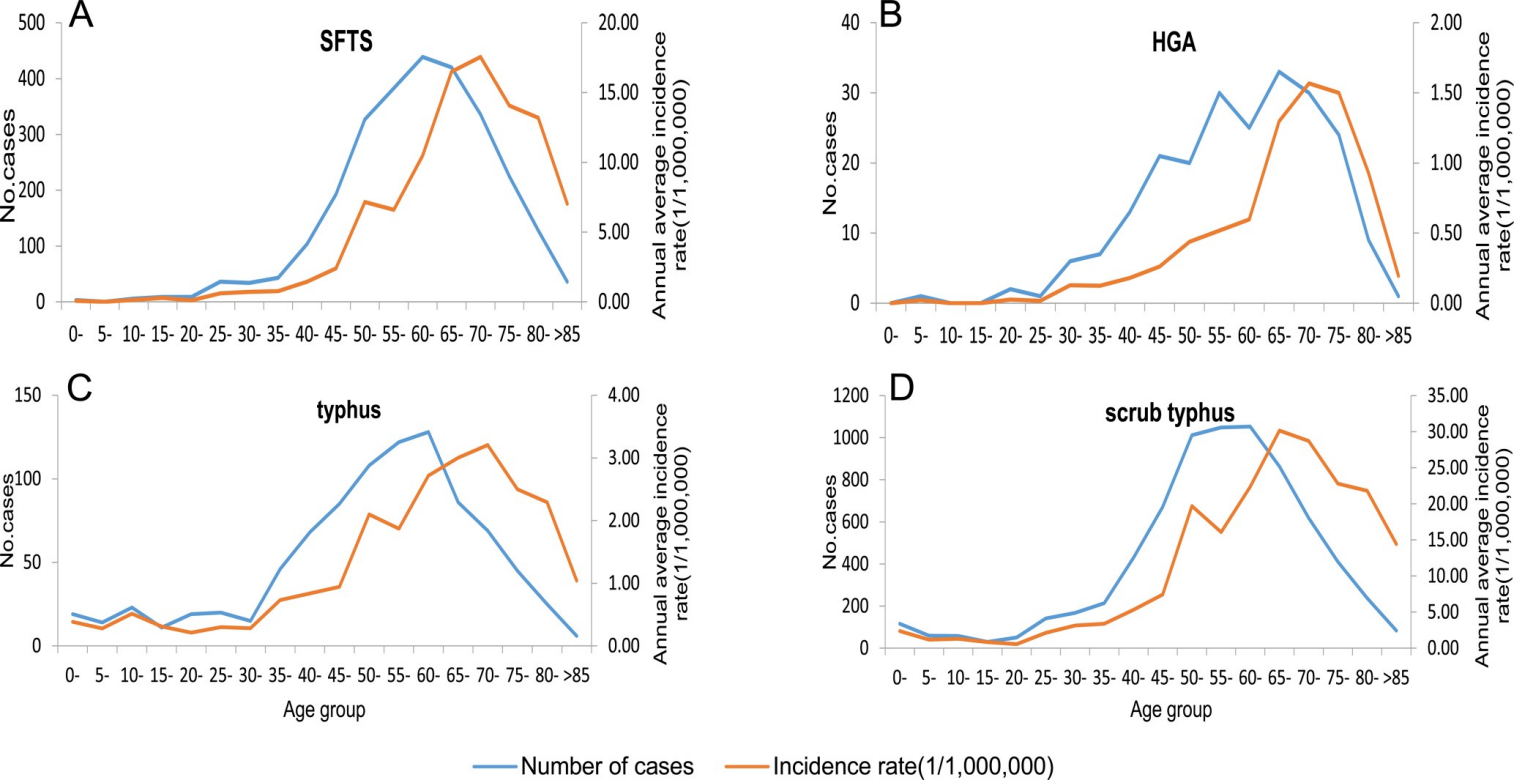
Age group distribution of the number of cases and incidence rate in Shandong Province, 2009–2017. (A): Age group distribution of SFTS cases and incidence rate. (B): Age group distribution of HGA cases and incidence rate. (C): Age group distribution of typhus cases and incidence rate. (D): Age group distribution of scrub typhus cases and incidence rate.

Of the total SFTS cases, 1384 cases were male and 1347 cases were female, the male-to-female ratio was 1.03: 1, and while there were slightly more male cases than female cases, the difference was not statistically significant (*χ*^2^ = 0.012, *P* = 0.924) (Table 1). The majority of SFTS cases were farmers (86.01%, 2349/2731) and the occupation distribution of SFTS cases in different years were similar (Table 1; Fig 4A). 94.87% (2591/2731) of SFTS cases occurred in individuals aged over 40 years (Table 1). The highest peak of the age group distribution of the number of SFTS cases occurred in the 60–65 age group. However, the highest peak of the age group distribution of the SFTS incidence rate lagged 2 age groups behind and appeared in the 70–75 age group (Fig 5A).

#### Regional distribution

**Fig 6:**
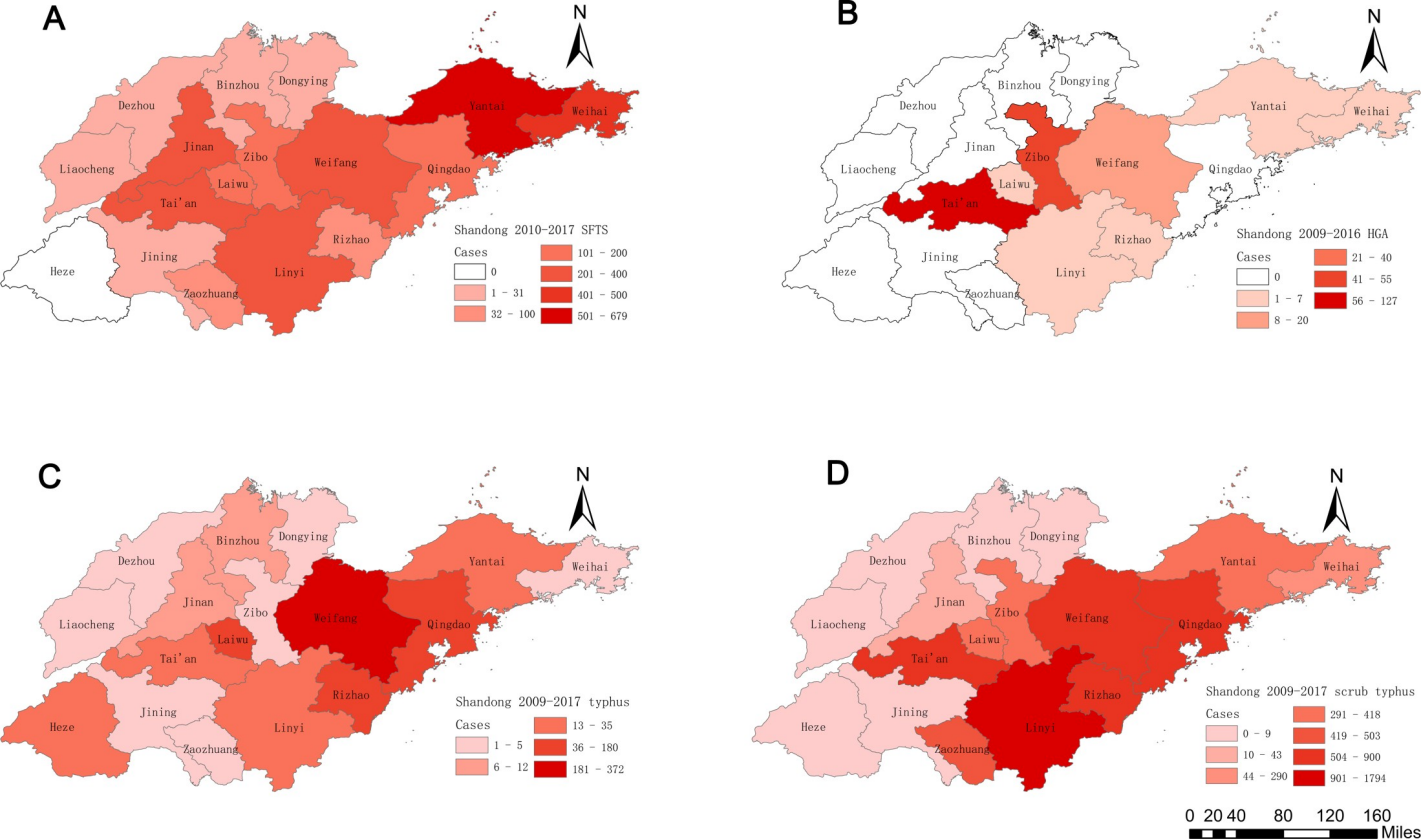
The geographic distribution of the four natural-focal diseases cases in Shandong Province, 2009–2017. (A): Geographical distribution of SFTS cases in Shandong Province. (B): Geographical distribution of HGA cases in Shandong Province. (C): Geographical distribution of typhus cases in Shandong Province. (D): Geographical distribution of scrub typhus cases in Shandong Province.

During 2010–2017, 86.34% of SFTS cases were limited to 7 of 17 cities in Shandong Province: Yantai (24.86%, 679/2731), Weihai (16.33%, 446/2731), Tai’an (12.45%, 340/2731), Jinan (11.09%, 303/2731), Weifang (8.75%, 239/2731), Linyi (7.54%, 206/2731), Qingdao (5.31%, 145/2731). In addition to these 7 cities, other cities reported only 373 SFTS cases (Table 2). Of note, 46.50% (1270/2731) of SFTS cases occurred on the Shandong Peninsula (including Yantai, Weihai and Qingdao). Furthermore, no case was reported in Heze in 2010 to 2017 (Table 2, Fig 6A). The numbers of affected cities in 2010 to 2017 were 5, 11, 11, 15, 13, 14, 13, and 14, respectively (S1 Table).

**Table 2:** Regional distribution of the four of natural-focal diseases in Shandong Province, 2009–2017.

	SFTS (2010–2017)		HGA (2009–2016)		Typhus (2009–2017)		Scrub typhus(2009–2017)	
	No. of cases	Proportion(%)	No. of cases	Proportion(%)	No. of cases	Proportion(%)	No. of cases	Proportion(%)
City								
Qingdao	145	5.31	0	0.00	99	10.89	835	11.50
Yantai	679	24.86	7	3.14	22	2.42	359	4.94
Weifang	239	8.75	20	8.97	372	40.92	815	11.23
Weihai	446	16.33	4	1.79	1	0.11	290	3.99
Rizhao	66	2.42	4	1.79	106	11.66	900	12.40
Linyi	206	7.54	2	0.90	30	3.30	1794	24.71
Liaocheng	1	0.04	0	0.00	1	0.11	1	0.01
Laiwu	117	4.28	4	1.79	180	19.80	418	5.76
Dezhou	11	0.40	0	0.00	1	0.11	0	0.00
Dongying	7	0.26	0	0.00	3	0.33	1	0.01
Zibo	118	4.32	55	24.66	1	0.11	390	5.37
Binzhou	8	0.29	0	0.00	12	1.32	8	0.11
Zaozhuang	38	1.39	0	0.00	5	0.55	503	6.93
Jining	7	0.26	0	0.00	2	0.22	9	0.12
Heze	0	0.00	0	0.00	35	3.85	1	0.01
Jinan	303	11.09	0	0.00	7	0.77	43	0.59
Tai'an	340	12.45	127	56.95	32	3.52	893	12.30

#### Distribution of fatal cases

A total of 251 deaths were reported in Shandong Province from 2010 to 2017 and the fatality rate was 9.19%, with the highest recorded in 2011 (13.69%, 23/168) and the lowest in 2016 (7.00%, 44/629). The fatality rate declined with the year (Mantel-Haenszel test of trend, χ^2^ = 11.823, *P* = 0.001). The fatality rate of males (10.04%, 139/1384) was slightly higher than females (8.61%, 116/1347), but the difference was not statistically significant (*χ*^2^ = 1.653, *P* = 0.212). The fatality rates of laborers, retirees, farmers, and others were respectively 11.76% (6/51), 10.39% (8/77), 9.49% (223/2349), and 5.91% (14/237). All fatal SFTS cases were observed in people aged over 35, and the fatality rates were higher in older age groups (Mantel-Haenszel test of trend, *χ*^2^ = 43.920, *P*<0.001), except that the fatality rates were low in the age groups of over 85. In the 11 cities with SFTS deaths, there was a significant difference in the fatality rate (χ^2^ = 86.569, P<0.001). Dezhou had the highest fatality rate (18.18%, 2/11) and Weifang had the lowest (0.84%, 2/239), no deaths were reported in the other 6 cities (Table 3).

**Table 3:** Demographic and epidemiological characteristics of fatal cases of the four natural-focal diseases in Shandong Province, 2009–2017.

	SFTS (2010–2017)		HGA (2009–2016)		Typhus (2009–2017)		Scrub typhus(2009–2017)	
	No. of deaths	Fatality rate(%)	No. of deaths	Fatality rate(%)	No. of deaths	Fatality Rate(%)	No. of deaths	Fatality rate(%)
Age group (years)								
0-	0	0.00	0	0.00	0	0.00	0	0.00
35-	4	2.72	0	0.00	0	0.00	0	0.00
45-	21	4.04	0	0.00	0	0.00	0	0.00
55-	77	9.37	2	3.64	0	0.00	1	0.05
65-	94	12.43	2	3.17	0	0.00	0	0.00
75-	53	15.01	1	3.03	0	0.00	0	0.00
>85	2	5.56	0	0.00	0	0.00	0	0.00
Total	251	9.19	5	2.24	0	0.00	1	0.01
Gender								
Male	139	10.04	5	4.39	0	0.00	0	0.00
Female	116	8.61	0	0.00	0	0.00	1	0.02
Occupation								
Children	0	0.00	0	0.00	0	0.00	0	0.00
Students	0	0.00	0	0.00	0	0.00	0	0.00
Laborers	6	11.76	0	0.00	0	0.00	0	0.00
Farmers	223	9.49	4	2.15	0	0.00	0	0.00
Retirees	8	10.39	1	8.33	0	0.00	0	0.00
Others	14	5.91	0	0.00	0	0.00	1	0.33
Year								
2009	-	-	1	1.92	0	0.00	0	0.00
2010	6	10.53	2	1.71	0	0.00	0	0.00
2011	23	13.69	1	5.88	0	0.00	1	0.22
2012	17	12.78	1	12.50	0	0.00	0	0.00
2013	25	8.45	0	0.00	0	0.00	0	0.00
2014	58	13.33	0	0.00	0	0.00	0	0.00
2015	41	8.23	0	0.00	0	0.00	0	0.00
2016	44	7.00	0	0.00	0	0.00	0	0.00
2017	37	7.18	-	-	0	0.00	0	0.00
City								
Dezhou	2	18.18	0	0.00	0	0.00	0	0.00
Yantai	99	14.58	0	0.00	0	0.00	0	0.00
Jinan	39	12.87	0	0.00	0	0.00	0	0.00
Tai'an	43	12.65	4	3.14	0	0.00	0	0.00
Qingdao	14	9.66	0	0.00	0	0.00	0	0.00
Zibo	9	7.63	0	0.00	0	0.00	0	0.00
Weihai	29	6.50	0	0.00	0	0.00	0	0.00
Rizhao	4	6.06	0	0.00	0	0.00	0	0.00
Laiwu	4	3.42	0	0.00	0	0.00	0	0.00
Linyi	6	2.91	0	0.00	0	0.00	1	0.06
Weifang	2	0.84	1	5.00	0	0.00	0	0.00
Other	0	0.00	0	0.00	0	0.00	0	0.00

Note: “-” represents lack of data.

### HGA

From 2009 to 2016, a total of 223 confirmed cases of HGA including 5 deaths in Shandong Province were reported to SDRIS.

#### Time distribution

During 2009–2016, an average of 0.29 cases per one million residents each year were reported, with the highest recorded in 2010 (1.22 cases/1,000,000) and the lowest in 2016 (0.01 cases/1,000,000) (Fig 2). The incidence of HGA had obvious seasonal characteristics, with 94.62% (211/223) of the HGA cases clustering between May and October. The peaks of the reported HGA cases in 2009–2016 occurred in the summer and autumn—July, August, August, August, June, July, and June, respectively (Fig 3B; Table 1).

#### Population distribution

Of the total HGA cases, 114 cases were males and 109 cases were females, and the male-to-female ratio was 1.05: 1. While there were slightly more male cases than female cases, the difference was not statistically significant (χ^2^ = 0.027, P = 0.984) (Table 1). The majority of HGA cases were farmers (83.41%, 186/223) (Table 1; Fig 4B). 92.38% (206/223) of the HGA cases occurred in individuals aged over 40 years old (Table 1). The highest peak of the age group distribution of the number of HGA cases occurred in the 65–70 age group. However, the 65–70 age group had more HGA cases compared to other age groups. The 70–75 age group showed the highest HGA incidence rate (Fig 5B).

#### Regional distribution

During 2009–2016, 90.58% of HGA cases were limited to 3 of 17 cities in Shandong Province: Tai’an (56.95%, 127/223), Zibo (24.66%, 55/223), and Weifang (8.97%, 20/223). In addition to these 3 cities, 21 HGA cases were reported in other cities (Table 2; Fig 6B). The numbers of affected cities in 2009 to 2016 were 5, 8, 2, 2, 3, 4, 1 and 1, respectively (S1 Table).

#### Distribution of fatal cases

A total of 5 deaths were reported in Shandong Province from 2009 to 2016 and the fatality rate was 2.24%. 1 case occurred in 2009, 2 cases occurred in 2010, 1 case occurred in 2011, and 1 case occurred in 2012. The 5 fatal cases were all male. The fatality rate of males (4.39%, 5/114) was higher than females (0.00%, 0/109), but the difference was not statistically significant (χ^2^ = 3.094, P = 0.079). Among the 5 fatal cases, one was a retiree and the other 4 were farmers. All HGA death cases occurred in individuals aged over 55 years old. Only 2 cities had fatal cases, the fatality rate of Tai’an was 3.14% (4/127) and Weifang was 5% (1/20) (Table 3).

### Typhus

From 2009 to 2017, a total of 909 confirmed cases of typhus in Shandong Province were reported to SDRIS.

#### Time distribution

During 2009–2017, an average of 1.04 cases per one million residents each year was reported, with the highest recorded in 2014 (1.49 cases/1,000,000) and the lowest in 2010 (0.70 cases/1,000,000) (Fig 2). The incidence of typhus had obvious seasonal characteristics, 81.19% (738/909) of cases occurred in autumn and winter, and 69.31% (630/909) of cases occurred in October and November, and peaked in October each year (Fig 3C; Table 1).

#### Population distribution

Of the total typhus cases, 482 cases were males and 427 cases were females, and the male-to-female ratio was 1.13: 1. While there were slightly more male cases, the difference was not statistically significant (χ^2^ = 2.184, P = 0.144) (Table 1). The majority of typhus cases were farmers (85.04%, 773/909) (Table 1; Fig 4C). All age groups were susceptible to typhus, 81.63% (742/909) of the typhus cases occurred in individuals aged over 40 years old (Table 1). The highest peak of the age group distribution of the number of typhus cases occurred in the 60–65 age group. However, the highest peak of the age group distribution of the typhus incidence rate lagged 2 age groups behind and appeared in the 70–75 age group (Fig 5C).

#### Regional distribution

During 2009–2017, 83.28% of typhus cases were limited to 4 of 17 cities in Shandong Province: Weifang (40.92%, 372/909), Laiwu (19.80%, 180/909), Rizhao (11.66%, 106/909), and Qingdao (10.89%, 99/909). In addition to these 4 cities, only 152 typhus cases were reported in other cities (Table 2; Fig 6C).The numbers of affected cities in 2009 to 2017 were 10, 10, 8, 11, 10, 9, 13, 8 and 11, respectively (S1 Table).

### Scrub Typhus

From 2010 to 2017, a total of 7260 confirmed cases of scrub typhus including 1 death in Shandong Province were reported to SDRIS.

#### Time distribution

During 2009–2017, an average of 8.21 cases per one million residents were reported each year, with the highest recorded in 2014 (15.27 cases/1,000,000) and the lowest in 2009 (2.40 cases/1,000,000) (Fig 2). The incidence of scrub typhus had obvious seasonal characteristics, 98.20% (7129/7260) of cases occurred in autumn and winter, and 97.06% (7047/7260) of cases occurred between September and November, with the highest peak in October (Fig 3D; Table 1).

#### Population distribution

Of the total scrub typhus cases, 3148 cases were males and 4112 cases were females. The male-to-female ratio was 0.77: 1, and the difference was statistically significant (χ^2^ = 151.16, P<0.001) (Table 1). The majority of scrub typhus cases were farmers (88.43%, 6420/7260) (Table 1, Fig 4D). All age groups were susceptible, and 88.53% (6427/7260) of typhus cases occurred in individuals aged over 40 years old (Table 1). The 60–65 age group had more scrub typhus cases compared to the other age groups but had a lower incidence rate than the 65–70 age group which showed the highest incidence rate among the groups (Fig 5D).

#### Regional distribution

During 2009–2017, scrub typhus cases were widespread in most of the cities in Shandong Province, 72.13% of scrub typhus cases were limited to 5 of 17 cities in Shandong Province: Linyi (24.71%, 1794/7260), Rizhao (12.40%, 900/7260), Tai’an (12.30%, 893/7260), Qingdao (11.50%, 835/7260), and Weifang (11.23%, 815/7260). 2023 scrub typhus cases were reported in other cities (Table 2; Fig 6D). The numbers of affected cities in 2009 to 2017 were 11, 12, 13, 12, 11, 13, 12, 13 and 13, respectively (S1 Table).

#### Distribution of fatal cases

A female from the 55–65 year old group in Linyi died from scrub typhus in 2011. This was the only fatal case reported during 2009–2017 (Table 3).

## Discussion

In this study, passive surveillance data of four natural-focal diseases were used to describe the epidemic characteristics of natural-focal diseases from 2009 to 2017 in Shandong Province, northern China.

### Annual distribution

The results showed that the number of cities with SFTS cases increased rapidly from 5 in 2010 to 14 in 2017, and the numbers of SFTS cases rose from 2010 to 2017 except in 2012 and 2017. Three factors may contribute to these results. First, since the first discovery of SFTSV in China in 2009, doctors and health departments have been more and more aware of SFTS, and missed diagnosis or misdiagnosis was reduced [1]. Second, SFTSV might have spread to more areas through humans, ticks, small mammals, or birds [6, 11]. Therefore, more people had the opportunity to be infected with SFTSV. Third, with the advancement of China's new rural construction and urbanization, the opportunities for ticks to contact people increased. A slight decline in the number of SFTS cases in 2012 and 2017 may be explained by different reasons. In 2012, only two years of monitoring of SFTSV in China were conducted. Doctors in various towns and counties may have insufficient understanding of SFTS, which may lead to missed diagnosis [12]. Therefore, the number of reported SFTS cases may not be accurate. After the health department explained the various hazards and prevention measures of SFTS to villagers in SFTS epidemic area, villagers began to use drugs to kill ticks biting livestock. These preventive and control measures began to work, so the number of SFTS patients decreased in 2017. We will continue to conduct long-term monitoring in the future to evaluate the long-term effects of these measures. In a comparison of the annual average incidence of Shandong Province and the annual average incidence from 2011 to 2016 of China, the incidence of Shandong Province (3.47 cases/1,000,000) was far higher than the national level (0.65 cases/1,000,000) [12]. The results showed that Shandong Province was an important epidemic area of SFTS in China. However, the reasons for this result need to be further studied.

Shandong Province is an emerging epidemic focus of HGA, as the first confirmed case was found in 2008 [13]. During 2009–2016, the number of cities with HGA cases decreased from 8 in 2010 to 1 in 2016, and the number of reported cases of HGA reached the highest in 2010, then sharply declined. This may be related to the following factors. First, before 2010, many SFTS patients were misdiagnosed as HGA [14]. With the discovery of SFTSV and the improvement of SFTS diagnostic methods, the incidence of HGA tends to be accurate. Second, as far as we know, because there had been few cases in the past few years, the health departments reduced its attention to HGA.

The incidence of typhus in Shandong Province has been very high. From 1994 to 2003, 6653 cases of typhus occurred, accounting for 14.10% (6653/47145) of the total number of cases in China [7]. However, the number of typhus cases in Shandong Province has dramatically decreased, with 909 cases occurring during 2009–2017, none of them were fatal, and the number of cities with typhus cases was relatively stable, about 10 per year in 2009–2017. This may be related to the improvement of people's living environment and health habits in recent years, such as rural toilet improvement, garbage sorting and recycling, river pond treatment, and other ecological environmental improvement work in rural areas of Shandong Province, which may have reduced the chances of people’s direct contact with *X*.*cheopis* and rodents. In addition, studies showed that typhus can spread by international travel [15, 16]. With an increasing number of Chinese tourists abroad, it is likely that there will be cases of imported typhus in China, which should attract the attention of the customs department. The number of cases of typhus in 2009–2017 was relatively stable, which may be related to the passive monitoring strategy of the Shandong Center for Disease Control and Prevention (Shandong CDC). According to our understanding, the passive monitoring strategy mainly relies on qualified units (hospitals and CDCs) to conduct direct network reporting. Due to the low fatality rate of this disease, it has been neglected by many units, and the units that have reported on the initiative are relatively few and fixed, so the number of cases of typhus was relatively stable.

Shandong Province is an emerging epidemic focus of scrub typhus, as the number of reported cases has rapidly increased since the first cases reported in 1986 [17–20]. The present study showed that the number of cities with scrub typhus cases was relatively stable and the number of annual reported cases of scrub typhus slowly increased before 2013, but there was a sharp increase from 2013 to 2014 and then it remained at a high level. This may be related to the following factors. First, Shandong CDC conducted active surveillance of scrub typhus in some parts of five cities (Linyi, Zibo, Tai'an, Qingdao, and Laiwu) from April 2013 to December 2015, which may have caused a sharp increase in the number of reported scrub typhus cases after 2013. Second, the availability of detection facilities because of the increasing investment of health resources may have affected the number of annual reported cases.

### Monthly and seasonal distribution

The four natural-focal diseases all had obvious seasonal characteristics. SFTS and HGA mainly occurred in summer and autumn in Shandong Province, with the epidemic peak in May to August, which was similar to other SFTS epidemic areas, such as those in Henan, Anhui, Jiangsu, and Zhejiang Provinces [21–23]. *Haemaphysalis longicornis* is the main tick species in Shandong Province, and SFTSV and AP are believed to be mainly transmitted by *H*.*longicornis* tick bites [6, 24]. Due to the lack of data on tick density fluctuations, we referred to two adjacent provinces, Henan and Jiangsu, which have a similar geographical location, climate type, and tick species distribution as Shandong Province. The peak of tick density in Henan and Jiangsu occurs from May to August, and we speculated that the peak of tick density in Shandong Province also occurs from May to August [25, 26]. Therefore, we believe that the epidemic peaks of SFTS and HGA are consistent with the fluctuation of tick density in Shandong Province.

Typhus mainly occurs in autumn and winter in Shandong Province, with the epidemic peak in October to November, which is different from other typhus epidemic areas, such as those in Henan, Yunnan, and Zhejiang Provinces [27–29]. The reason for this may be that Shandong Province, with a lower temperature, is located in the north of these epidemic areas, and thus the main vector of *X*. *cheopis* would reach a peak later. In addition, October and November are the harvest time in Shandong Province, and farmers have frequent contacts with the *X*. *cheopis* which may have resulted in an increase in the number of cases.

Scrub typhus mainly occurs in autumn and winter in Shandong Province, with the epidemic peak in September to November, which may be associated with the following factors. First, September to November is the harvest time in Shandong Province, with outdoor activities of farmers increasing during this period and enhances the risk for farmers to come in contact with the main transmission vector of *Leptotrombidium scutellare* [30]. Second, rodents and *L*.*scutellare* are the main reservoir hosts and transmission vectors for *Orientia tsutsugamushi* in Shandong Province. Their density fluctuations are closely related to the seasonal distribution of scrub typhus cases, and the monthly distribution of scrub typhus cases is consistent with the fluctuation of *L*. *scutellare* [8, 31, 32]. Third, some studies have shown that meteorological factors affect the incidence of scrub typhus, such as temperature, sunlight, and precipitation [17, 33, 34]. The climatic conditions of Shandong Province from September to November may be most suitable for the occurrence of scrub typhus. The above factors may contribute to the high infection rate of scrub typhus during autumn.

### Population distribution

The high-risk groups of the four diseases are all farmers and the elderly. This may be related to the following factors. First, with the advancement of China's urbanization process and the expansion of enrollment in higher education institutions, a large number of middle-aged men and young people from rural areas have moved to cities to work or study, and the elderly become the main force of agricultural production [35]. Therefore, the elderly are more frequently exposed to arthropod vectors during their agricultural activities than young people who work or study in cities apart from the Spring Festival (also known as Chinese or Lunar New Year) when they visit their homes in rural areas [36]. Second, the immune function of the elderly may be lower than young people. If they are infected with these four diseases, the elderly may get severe cases and go to hospital for treatment [37, 38]. Therefore, more elderly cases were identified.

The highest peaks of the age group distribution of the incidence rates of the four diseases lagged behind the highest peak of the age group distribution of the number of cases by 1 or 2 age groups. The 60–65 age group had the highest number of cases (except for HGA), but the 70–75 age group had the highest incidence rate (except for scrub typhus). This is related to the age structure of the population in Shandong Province. Compared with the 60–65 age group, the 70–75 age group has a smaller population, so the incidence rate is higher. This may also reflect that the 70–75 age group is the highest risk group for natural-focal diseases in Shandong Province.

The results showed that the incidence of scrub typhus among females was higher than that in males, which was consistent with previous studies [18, 19]. Two factors may account for the result- the increased proportion of females engaging in outdoor activities [39], and the higher susceptibility of females to *O*. *tsutsugamushi* compared with males [40] which may require further investigation and experimentation.

### Regional distribution

SFTS and HGA cases in Shandong Province were mainly reported in the middle-southern part of the Province (Linyi, Jinan, Tai’an, Weifang, Zibo, and Laiwu) and the Shandong Peninsula (Qingdao, Yantai, and Weihai). The density of ticks may be higher in the mountains areas which are abundant in shrub grasslands, so there were more SFTS and HGA cases [41]. Therefore, the mountainous areas with high vegetation cover should be the key areas for SFTS and HGA prevention and control.

Typhus cases in Shandong Province were mainly concentrated in inland mountainous areas (Laiwu) and coastal areas (Rizhao, Qingdao, and Weifang). The incidence of typhus cases may be related to rural the rodent density and *X*.*cheopis* density in these areas, but the relevant survey data are lacking at present. Subsequent studies can further explore the factors affecting the distribution of typhus cases in different regions by investigating the density of rural rodents and *X*.*cheopis* density in different regions.

Most scrub typhus cases in Shandong Province were concentrated in inland mountainous areas (Linyi and Tai’an) and coastal hilly areas (Rizhao, Qingdao, and Weifang), which could be partly explained by the geographical distribution and population density of chigger mites [42]. It is possible that chigger mites are more abundant in the low mountains and hills. The higher vegetation cover in the low mountains and hills, overgrown weeds, suitable temperature, abundant precipitation, and humid environment are suitable for the survival and reproduction of chigger mites.

### Distribution of fatal cases

All SFTS fatal cases occurred in individuals aged over 35 years, and laborers, retirees, and farmers had higher fatality rates. Two factors might contribute to this. First, most of the cases aged 35 were farmers and laborers whose medical conditions were poor in rural areas or the construction site where they lived might have caused the diagnosis and treatment to be delayed. Combined studies showed that the time spent before diagnosis could affect the prognosis [43]. Second, retirees are elderly, and once infected by SFTSV, the elderly might have severe cases [37]. So the fatality rate was higher than that of mild or even asymptomatic patients.

Previous studies showed that the fatality rate of SFTS increases with age [12, 44]. Our study also indicated that age was closely related to the SFTS fatality rate. The decrease in fatality in the age group of over 85 may be due to the fact that the population of people aged over 85 was small and their deaths caused by SFTS were rare cases. Some other factors associated with age including the weakened immune function and that comorbidities with chronic diseases may be linked to the fatal outcome of SFTS cases.

The fatality rate of SFTS decreased from 2010 to 2017, but it remained very high in 2017(7.18%). The high fatality rate suggested that it is urgent to develop effective vaccines and treatments for SFTS. The decline of the fatality rate might be explained by improvement of diagnosis and treatment of SFTS cases [12].

The fatality rate of SFTS varied in different regions. This may be related to the following factors. First, the proportions of severe cases in different regions might were different, but the lack of relevant data is important. Second, there were differences in the medical level and doctor's experience. Based on what we know, we believe that the latter is more likely to be the main reason.

All HGA fatal cases occurred in individuals aged over 55 years. Two factors may account for the result. First, most of the cases were farmers, and the medical conditions were poor in rural areas where their diagnosis and treatment may have been delayed [45]. Second, it may be related to the low immune function of the elderly and even worse therapeutic effects [38, 45]. The average case fatality rate was 2.24% in Shandong Province, which was higher than the rate of 0.3% in the United States [46]. This may be due to the following two facts. First, HGA was still not recognized by many Chinese doctors at that time, which was likely to cause misdiagnosis or delayed antibiotic therapy [47, 48]. Second, the outer membrane protein msp2/p44 as important virulence factors of AP pathogens, and their molecular characteristics are significantly different in strains isolated from China and the United States at the nucleic acid, amino acid, and protein levels [49]. Therefore, Chinese patients often showed more serious clinical manifestations and a higher mortality rate [49].

The five fatal cases of HGA all occurred in 2009–2012, which may be related to the fact that many SFTS were misdiagnosed as HGA during this time, thus leading to the emergence of fatal cases [14, 47].

Although only one scrub typhus fatal case occurred in Shandong Province from 2009 to 2017, we should not relax our vigilance in the treatment of patients with scrub typhus.

## Limitations

There are some limitations in our study. First, since the case data were obtained from a passive surveillance system, the reporting system might have missed some cases because some patients had no clinical manifestations or only mild performance, or had not been diagnosed or treated in hospitals. In addition, there is no equipment or technology in many grassroots areas to diagnose and identify the pathogens of natural-focal diseases. Thus the number of reported cases was much smaller than the actual number of cases. Second, misdiagnosis and lack of awareness of some of the reported species of natural-focal diseases by some Chinese doctors may result in fewer reported cases than actual cases. Third, due to the limited data, the four natural-focal diseases described above cannot fully represent the natural-focal diseases in Shandong Province. Fourth, the residential address information of the reported cases was only accurate to the city-level. If the information was accurate to the county or even town levels, we could describe the geographical distribution of the cases at a higher resolution. This is more conducive to the accurate allocation of resources.

## Conclusions

Despite the limitations stated above, our study described the epidemic characteristics, and identified spatiotemporal clusters of four natural-focal diseases (SFTS, typhus, scrub typhus, and HGA) in Shandong Province during 2009–2017. Our findings can contribute to effective allocation of resources by public health officials for the prevention and control of natural-focal diseases in Shandong Province.

## Supporting information

S1 Checklist STROBE checklist. (DOCX)

S1 File Diagnosis criteria of four natural-focal diseases. (DOCX)

S1 Table The number of cities affected by four natural-focal diseases, 2009–2017. (XLSX)
