# Supplementary material for: Analysis of epidemiological characteristics of four natural-focal diseases in Shandong Province, China in 2009-2017: A descriptive analysis
Source: PLoS One. 2019 Aug 27;14(8):e0221677. doi: 10.1371/journal.pone.0221677 (PMC6711524; doi:10.1371/journal.pone.0221677)
Supplement: S1 File — (DOCX) [file pone.0221677.s002.docx]

**SFTS**

[http://www.moh.gov.cn/mohwsyjbgs/s8348/201010 /49272.shtml](http://www.moh.gov.cn/mohwsyjbgs/s8348/201010%20/49272.shtml).

**Case definition.** An acutely ill person with acute onset of fever (≥38.0°C) and other symptoms (e.g. gastrointestinal symptoms, bleeding), epidemiological risk factors (being a farmer or being exposed to ticks two weeks before illness onset)and laboratory data consisting of thrombocytopenia and leukocytopenia was defined as a probable case. A probable case with one or more of the following criteria: (1) detection of SFTSV RNA, (2) seroconversion or 4-fold increase in antibody titers between paired serum samples collected at least two weeks apart, and (3) isolation of SFTSV in cell culture was defined as a confirmed case.

**Typhus**

<http://www.nhfpc.gov.cn/zwgkzt/s9491/200802/38814.shtml>

**epidemic typhus**

**Case definition.** The criteria for a probable case include acute persistent fever and epidemiological risk factors (cases often occur in winter and spring, patients often have body louse on their clothes or clothes). A probable case with one or more of the following criteria: (1) symptoms of rash (occurred first in the armpit and two ribs, followed by the most obvious back), (2) nervous system symptoms (e.g. headache, restlessness and lethargy) was defined as a clinical diagnosis case. A probable case or clinical diagnosis case with one or more of the following criteria: (1) an agglutination titer ≥1:160 in the Weil-Felix test using the OX_19_ strain, and the agglutination titer of OX_19_ strain increased 4-fold or over 4-fold with the course of disease, (2) serum antibody titer of Rickettsia przewalskii IgM≥1:40 or IgG≥1:160, or the antibody titer of the two serum samples increased by 4-fold or more by indirect immunofluorescence assay (IFA), (3) detection of Rickettsia przewalskii DNA, (4) isolation of Rickettsia przewalskii was defined as a confirmed case.

**endemic typhus**

**Case definition.** The criteria for a probable case include acute persistent fever and epidemiological risk factors (cases often occur in autumn and winter, patients often have flea contact history or live where there are many rats). A probable case with symptoms of rash was defined as a clinical diagnosis case. A probable case or clinical diagnosis case with one or more of the following criteria: (1) an agglutination titer ≥1:160 in the Weil-Felix test using the OX19 strain, and the agglutination titer of OX19 strain increased 4-fold or over 4-fold with the course of disease, (2) serum antibody titer of Rickettsiae Mooseri IgM≥1:40 or IgG≥1:160, or the antibody titer of the two serum samples increased by 4-fold or more by indirect immunofluorescence assay (IFA), (3) detection of Rickettsiae Mooseri DNA, (4) isolation of Rickettsiae Mooseri was defined as a confirmed case.

**Scrub typhus**

<http://www.chinacdc.cn/tzgg/200901/t20090105_40316.html>

**Case definition.** The criteria for a probable case of scrub typhus include epidemiological exposure histories (traveling to an endemic area and contact with chiggers or rodents within three weeks before the onset of illness), clinical manifestations (such as high fever, lymphadenopathy, skin rash, and eschars or ulcers), and an agglutination titer ≥1:160 in the Weil-Felix test using the OXK strain of Proteus mirabilis. The case definition of confirmed scrub typhus must fulfill the above criteria for a probable case and also meet at least one of the laboratory criteria for confirmatory diagnosis: a 4-fold or greater rise in serum IgG antibody titers between acute and convalescent sera detected by using indirect immunofluorescence antibody assay (IFA), detection of O. tsutsugamushi by polymerase chain reaction (PCR) in clinical specimens, or isolation of O. tsutsugamushi from clinical specimens.

**HGA**

<http://www.moh.gov.cn/mohwsyjbgs/s6734/200804/31429.shtml>

**Case definition.** A probable case of HGA was defined in patients with a clinically compatible illness including epidemiology and typical manifestations similar to HGA and with the following results: Leukocyte, thrombocytopenia decreased, ALT and/or AST aminotransferase elevated.

A probable case with one or more of the following criteria: (1) a 4-fold change in antibody titer to Anaplasma species antigen by indirect immunofluorescence assay (IFA) in the acute and recovery phase serum samples, (2) detection of Anaplasma species DNA, (3) isolation and culture of Anaplasma species was defined as a confirmed case.
