# Supplementary material for: Analysis of epidemiological characteristics of four natural-focal diseases in Shandong Province, China in 2009-2017: A descriptive analysis
Source: PLoS One. 2019 Aug 27;14(8):e0221677. doi: 10.1371/journal.pone.0221677 (PMC6711524; doi:10.1371/journal.pone.0221677)
Supplement: Attachment — Submitted filename: Response to reviewers.docx [file pone.0221677.s004.docx]

Dear Editor,

We would like to thank the journal PLOS ONE for giving us the opportunity to revise our manuscript.

We thank the reviewers for their careful reading and thoughtful comments on our manuscript. We have carefully taken their comments into consideration in preparing our revision, which has resulted in a paper that is clearer, more compelling, and broader. The following summarizes how we responded to reviewer comments.

Below is our response to their comments.

Thanks for all the help.

Best wishes,

Dr. Wen Hongling

Revision — authors’ response

Introduction

Reviewer #1:

1. We accepted the advice of the reviewer and have started our manuscript with the definition of “natural-focal diseases” in the main text. (Line 56-57)

Methods

Reviewer #1:

1. We agreed with the reviewer's view that the reasons for choosing the four natural-focal diseases are inadequate in the manuscript. We choose the four natural-focal diseases for several reasons: First, the four natural-focal diseases are the key diseases monitored by the local health department because their great threat to local residents. HGA, typhus and scrub typhus are all rickettsial diseases, SFTS was misdiagnosed as HGA because of similar clinical manifestations before SFTSV was discovered. SFTS and HGA are emerging natural-focal diseases in Shandong Province, which are closely related each other and have high fatality rates. Typhus and scrub typhus are two natural-focal diseases with high morbidity in local areas. Second, the four natural-focal diseases are transmitted by insect vectors that live in similar ecological environments. Third, the main infected people of the four natural-focal diseases are farmers, and farmers are more likely to be ignored by people. For these reasons, we choose the four natural-focal diseases for description. The following is described in the manuscript: We selected four natural-focal diseases which had great impacts on the human health in Shandong Province. The four natural-focal diseases are HGA, typhus, SFTS, and scrub typhus, and are transmitted through insect vectors. SFTS and HGA are emerging natural-focal diseases in Shandong Province which are closely related to each other and have high fatality rates. Typhus and scrub typhus are two natural-focal diseases with high morbidity in local areas. The four natural-focal diseases were monitored intensively by the local health departments because of their great threat to local residents. (Line 76-82)

2. In line 118 of the manuscript, we explained the source of the data, as stated below: Information of the four natural-focal diseases cases includes gender, age, occupation, residential address, date of illness onset, and outcome of the illness comes from the Shandong Disease Reporting Information System (SDRIS). The SDRIS can only be accessed through the CDC Internal Control Network and authorization, so no citation was added.

3. We accepted the advice of the reviewer and have added citations of the statistical programs. (Line 125)

Results

Reviewer #1:

1. We accepted the advice of the reviewer and have deleted the relevant content of Figure 2A.

2. We have deleted the Chinese expression in Figure 3.

Conclusions

Reviewer #1:

We accepted the advices of the reviewer and revised some of the statements in the discussion section.

The following are the questions that the reviewer has listed:

1. We accept the advice of the reviewer, and the speculation that the number of SFTS cases in 2012 and 2017 is slightly reduced is too subjective and has no reference. By understanding the measures taken by the CDC staff to prevent SFTS in recent years, and the impact of their work on the villagers in SFTS epidemic area, we now re-estimate the possible reasons for the decline in the number of SFTS cases (the effects of these preventive measures were not published in the article). Described as follows: A slight decline in the number of SFTS cases in 2012 and 2017 may be explained by different reasons. In 2012, only two years of monitoring of SFTSV in China were conducted. Doctors in various towns and counties may have insufficient understanding of SFTS, which may lead to missed diagnosis [12]. Therefore, the number of reported SFTS cases may not be accurate. After the health department explained the various hazards and prevention measures of SFTS to villagers in SFTS epidemic area, villagers began to use drugs to kill ticks biting livestock. These preventive and control measures began to work, so the number of SFTS patients decreased in 2017. We will continue to conduct long-term monitoring in the future to evaluate the long-term effects of these measures. (Line 299-307)

2. We accepted the advice of the reviewer. We learned from the health department that the HGA monitoring strategy had changed (The relevant notices were communicated through internal documents and were not published on the web), and we described the speculative reasons as follows: Second, as far as we know, because there had been few cases in the past few years, the health departments reduced its attention to HGA. (Line 317-318)

3. We accepted the advice of the reviewer and revised line 327 as follows: With an increasing number of Chinese tourists abroad, it is likely that there will be cases of imported typhus in China, which should attract the attention of the customs department. (Line 328-330)

The following are the questions that the reviewer has not listed:

1. The following statements without reference support have been deleted:

1.1. Second, prevention and control measures of departments of health were effective. (Line 317-318)

1.2. Second, with the advancement of China's new rural construction and urbanization, the opportunities for chigger mites to contact people have increased. Third, the distribution range of the vector chigger mites and the main reservoir host rodents. (Line 343-345)

1.3. In addition, it should be noted that while SFTS and HGA cases were not reported in some areas this does not mean that the cases did not occur in these areas. The possible reasons might be that the patients’ clinical performances were not serious, or there were no medical diagnosis or a misdiagnosis might have been made. (Line 405)

2. Suitable references have been added to the following statements:

2.1 The decline of the fatality rate might be explained by improvement of diagnosis and treatment of SFTS cases [12]. (Line 434-435)

Editorial and Data Presentation

Reviewer #1: (No Response)

Summary and General Comments

Reviewer #1:

1. We accepted the advice of the reviewer and deleted the Ebola virus disease (EVD) in the manuscript. (Line 61)

2. We accepted the advice of the reviewer and described the sentences "Shandong Province is the second most populous province in China with a population of about 100 million, of which about 40% live on agricultural land." in the introduction section. (Line 66-68)
